# Pathological trajectory in the Ts65Dn model of Down syndrome

**DOI:** 10.18632/aging.204497

**Published:** 2023-01-20

**Authors:** Savannah Tallino, Wendy Winslow, Ramon Velazquez

**Affiliations:** 1 Arizona State University-Banner Neurodegenerative Disease Research Center at the Biodesign Institute, Arizona State University, Tempe, AZ 85281, USA; 2 School of Life Sciences, Arizona State University, Tempe, AZ 85281, USA; 3 Arizona Alzheimer’s Consortium, Phoenix, AZ 85014, USA

**Keywords:** Down syndrome, Ts65Dn, amyloid-beta, cholinergic

As the world’s population ages, dementia from Alzheimer’s disease (AD) is projected to increase astronomically; individuals with Down syndrome (DS) are counted among those virtually guaranteed to develop AD pathology and, if they live long enough, AD dementia [1, 2]. DS is characterized by intellectual disability and for decades featured a high incidence of congenital heart abnormalities leading to early mortality. However, recent medical advances drastically improved the life expectancy in DS; more than a quarter of a million people living with DS in the United States are now projected to age long enough to develop AD [2].

DS results from a triplication of the DS Critical Region of chromosome 21, which contains (among others) the gene for the amyloid precursor protein (APP). APP can be processed in an amyloidogenic manner when cleaved into soluble amyloid β (Aβ) fragments – predominantly Aβ_40_ and Aβ_42_ – that are neurotoxic and prone to oligomerization and aggregation into plaques [1]. The subsequent neuropathological hallmarks of sporadic AD and DS patients with AD are nearly identical, differing mainly in age of onset, with nearly all DS individuals showing Aβ plaques and neurofibrillary tangles of tau by age 40; additionally, DS males develop AD pathology at a younger age than females [1] but females experience a higher risk of death from AD than males [2]. It is now acknowledged that changes in the AD brain occur decades before clinical symptomology, where prodromal cellular alterations occurring before the accumulation of Aβ plaques and aberrant tau initiate a cascade of events, likely in a synergistic manner, that ultimately culminate in the neurodegeneration underlying cognitive deficits. The ramifications of this are clear – investigations of the earliest etiological stages in AD and DS with AD will help us understand *what* is going wrong in the AD brain and *why*, yielding better therapeutic targets and avenues for disease prevention.

One of the earliest changes in AD brains is the appearance of alterations to the endosomal-lysosomal network within basal forebrain cholinergic neurons (BFCNs) [1, 3]. BFCNs provide crucial cholinergic innervation to the hippocampus and prefrontal cortex for memory and executive functions (e.g., attention and planning), and the synapses connecting these regions are also affected by oligomeric Aβ [1]. Interestingly, BFCN loss correlates with cognitive decline, which is one reason why the earliest approved AD treatments were a class of drugs known as acetylcholinesterase inhibitors [1]. As in most cells, the endosomal-lysosomal network in BFCNs allows for endocytosis and subsequent processing and transport of a wide variety of molecules, as well as the degradation of unwanted cellular components within lysosomes [3]. Furthermore, studies have shown that neurotrophic growth factors such as neural growth factor (NGF), which are produced by their target regions, must bind the appropriate receptors on BFCNs, correctly traverse the endosomal-lysosomal system, and be transported up the long BFCN axons back to the soma [4]. There is evidence that APP degradation products (including Aβ and also cleavage products upstream of Aβ such as the β-C-terminal fragment) can interfere with this process at multiple stages (reviewed extensively in [4]), and previous work in DS models has shown that the presence of an extra APP gene alone is necessary for the early endosomal abnormalities seen in DS [3]. Conversely, it is also possible that alterations to the endosomal-lysosomal network of BFCNs can increase Aβ production within endosomes or impair the clearance of Aβ via autophagy within lysosomes, which is particularly relevant in DS where the additional copy of APP increases the likelihood of Aβ production along the amyloidogenic pathway. Understanding the interplay between soluble Aβ production and early changes to BFCNs and/or their target regions, and whether this process could set the brain on pathological trajectory, are thus crucial research questions, not only in DS and AD, but in other neurodegenerative disorders (e.g., Parkinson’s disease) which also show BFCN decline [1].

Mouse models are a valuable tool to study AD pathology mechanistically *in vivo*. However, mice do not spontaneously develop AD pathology observed in humans without the presence of mutations to amyloid- or tau-processing related genes (whether via humanized transgenes or knock-in mutations to murine genes). Additionally, unlike many AD mouse models, the segmentally trisomic Ts65Dn mouse model of DS [5] demonstrates BFCN pathology and loss. For example, trisomic mice show endosomal abnormalities as early as 2 months of age [3], followed by degeneration and loss of BFCNs around 6 months [6], that, as in humans, coincides with cognitive decline of the cholinergic-hippocampal system [7]. However, the timing of soluble Aβ accumulation coincident with the changes to brain regions such as the basal forebrain and hippocampus had not yet been clearly defined in DS or in the recently updated Ts65Dn mouse model (Jackson Laboratory Strain #005252).

**Figure 1:**
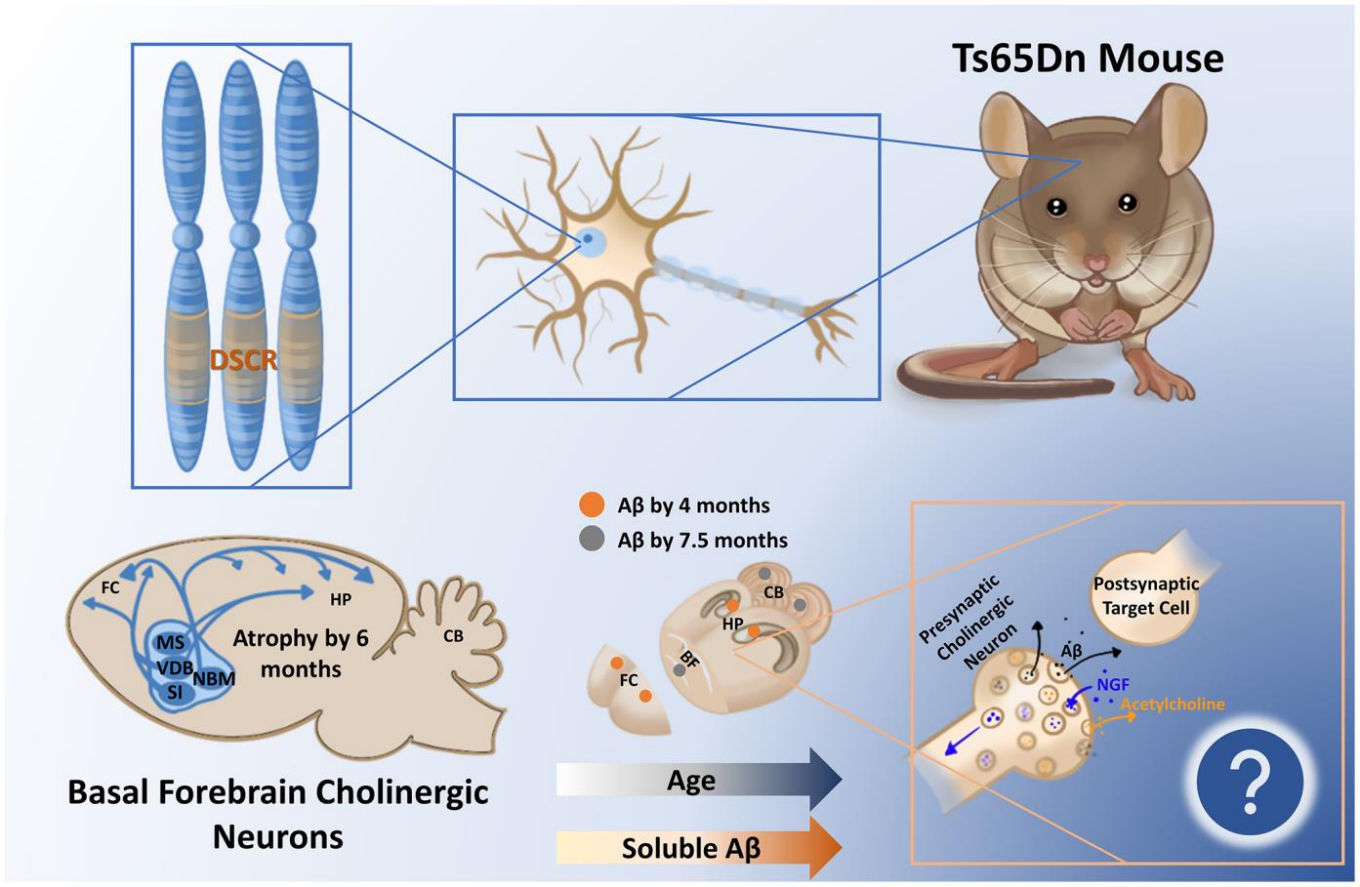
**The segmentally trisomic Ts65Dn mouse carries the majority of triplicated genes seen in human Down syndrome (DS) within the DS critical region (DSCR).** As a result of triplication of the amyloid precursor protein within the DSCR, soluble amyloid β (Aβ) increases in the Ts65Dn mouse brain as a function of age, appearing first in the frontal cortex (FC) and hippocampus (Hp). How the time-dependent regional changes in soluble Aβ relate to the documented atrophy of basal forebrain cholinergic circuitry at 6 months is yet to be determined but may relate to endosomal alterations and/or deficient nerve growth factor (NGF) transport in these vulnerable cells. Abbreviations: MS: medial septum; CB: cerebellum; VDB: ventral diagonal band; NBM: nucleus basalis of meynert; SI: substantia innominata.

In a recent publication, we analyzed soluble Aβ_40_ and Aβ_42_ in an age-and spatially-dependent manner in the Ts65Dn mouse model (Strain #005252) [8]. We collected basal forebrain, hippocampal, frontal cortical, and cerebellar brain tissue from male and female trisomic (3N) Ts65Dn mice and control (2N) littermates at 4, 7.5, and 12 months of age and assessed soluble Aβ_40_ and Aβ_42_ levels via enzyme-linked immunosorbent assay. We found that soluble Aβ_40_ and Aβ_42_ accumulate significantly in 3N animals in an age-dependent manner, starting in the frontal cortex and hippocampus by 4 months, followed by all examined brain regions by 7.5 months, and sustained as late as 12 months (Figure 1). Importantly, the accumulation of soluble Aβ_40_ and Aβ_42_ in the frontal cortex and hippocampus we observed by 4 months precedes the loss of BFCNs; it is possible this increase in soluble Aβ in these BFCN target regions affects retrograde NGF signaling back to the BFCN somas. Also, the significant elevation of Aβ_40_ and Aβ_42_ in the basal forebrain at 7.5 months of age coincides with the timing of previously-observed BFCN loss [6]. Another intriguing finding was that female 3N mice had significantly higher Aβ_40_ and Aβ_42_ than males in the basal forebrain at 7.5 months, which matches the sex-specific increases in BFCN loss at this age as reported previously [6]. Finally, we correlated the levels of soluble Aβ_40_ and Aβ_42_ with levels of full APP protein as determined via immunoblot. APP levels were significantly inversely correlated with soluble Aβ_40_ and Aβ_42_ levels, but only in the basal forebrain and hippocampus, suggesting that these two sites in particular may favor APP processing along the amyloidogenic pathway.

In conclusion, it is likely that the various hypotheses of AD etiology (cholinergic, amyloid cascade, tau, etc.) which have vied for preeminence across the past few decades instead require merging into a more holistic picture [4]. This synthesis may better describe how the production of soluble Aβ prior to plaque formation, and the coincident pathology in BFCNs, affects other neurodegenerative phenomena such as tau pathology and neuroinflammation. In DS specifically, we also need a better understanding of how the interplay between the many triplicated genes may influence the early onset of AD pathology within these vulnerable cells. Our findings in the Ts65Dn model (Strain #005252) will allow future work to assess the contribution of soluble Aβ accumulation within AD in the context of DS, as well as shedding light on the pathogenesis of BFCNs in neurodegenerative diseases in general.
